# Cortical circuit alterations precede motor impairments in Huntington’s disease mice

**DOI:** 10.1038/s41598-019-43024-w

**Published:** 2019-04-29

**Authors:** Johanna Burgold, Elena Katharina Schulz-Trieglaff, Kerstin Voelkl, Sara Gutiérrez-Ángel, Jakob Maximilian Bader, Fabian Hosp, Matthias Mann, Thomas Arzberger, Rüdiger Klein, Sabine Liebscher, Irina Dudanova

**Affiliations:** 1 Department of Molecules – Signaling – Development, Max Planck Institute of Neurobiology, 82152 Martinsried, Germany; 2 Department of Proteomics and Signal Transduction, Max Planck Institute of Biochemistry, 82152 Martinsried, Germany; 3 German Center for Neurodegenerative Diseases (DZNE), 81377 Munich, Germany; 4 Center for Neuropathology and Prion Research, Ludwig-Maximilians University Munich, 81377 Munich, Germany; 5 Department of Psychiatry and Psychotherapy, Ludwig-Maximilians University Munich, 81377 Munich, Germany; 6 Munich Cluster for Systems Neurology (SyNergy), 81377 Munich, Germany; 7 Institute of Clinical Neuroimmunology, Klinikum der Universität München, Ludwig-Maximilians University Munich, 82152 Martinsried, Germany; 8 Biomedical Center, Medical Faculty, Ludwig-Maximilians University Munich, 82152 Martinsried, Germany

**Keywords:** Motor cortex, Huntington's disease, Neural circuits

## Abstract

Huntington’s disease (HD) is a devastating hereditary movement disorder, characterized by degeneration of neurons in the striatum and cortex. Studies in human patients and mouse HD models suggest that disturbances of neuronal function in the neocortex play an important role in disease onset and progression. However, the precise nature and time course of cortical alterations in HD have remained elusive. Here, we use chronic *in vivo* two-photon calcium imaging to longitudinally monitor the activity of identified single neurons in layer 2/3 of the primary motor cortex in awake, behaving R6/2 transgenic HD mice and wildtype littermates. R6/2 mice show age-dependent changes in cortical network function, with an increase in activity that affects a large fraction of cells and occurs rather abruptly within one week, preceeding the onset of motor defects. Furthermore, quantitative proteomics demonstrate a pronounced downregulation of synaptic proteins in the cortex, and histological analyses in R6/2 mice and human HD autopsy cases reveal a reduction in perisomatic inhibitory synaptic contacts on layer 2/3 pyramidal cells. Taken together, our study provides a time-resolved description of cortical network dysfunction in behaving HD mice and points to disturbed excitation/inhibition balance as an important pathomechanism in HD.

## Introduction

Huntington’s disease (HD) is an incurable hereditary neurodegenerative disorder, characterized by choreatic movements in combination with cognitive decline and psychiatric symptoms. HD is caused by an expansion of the CAG repeat in exon 1 of the Huntingtin gene^1^, resulting in the expression of the aggregation-prone mutant Huntingtin (mHTT) protein with an elongated polyglutamine (polyQ) tract. mHTT interferes with multiple cellular functions, including transcription, energy metabolism, protein homeostasis and intracellular transport^2,3^. The striatum is the most vulnerable region in HD, however, prominent pathological changes are also observed in the neocortex^4–6^. Importantly, ample evidence points towards the disturbance of cortical function and impairment of corticostriatal communication as crucial early events in HD^7–10^. Imaging studies in human HD gene expansion carriers demonstrate that cortical thinning and abnormalities of cortical activity contribute to the onset, progression and clinical variability of the disease^6,11–16^. In particular, primary motor cortex (M1) is among the regions showing the earliest changes^15^, and the degree of cell loss in this area correlates with the motor impairments^17^. In addition, analyses of tissue-specific conditional mouse models revealed the requirement of mHTT in both the striatum and the cortex for driving the full extent of HD phenotypes^18,19^. Likewise, mHTT lowering in both regions is necessary for an efficient rescue of HD-related deficits^20,21^.

Previous studies in HD mouse models uncovered multiple morphological and electrophysiological abnormalities of cortical pyramidal neurons (principal cells, PCs). Reduced dendritic arborizations and a decline in the density and stability of dendritic spines on PCs were observed in the somatosensory cortex^22–24^. These defects were paralleled by lower levels of several synaptic proteins and a decrease in excitatory synapse density, detected at an advanced disease stage^24^. Moreover, conditional mouse models demonstrated the importance of mHTT expression in inhibitory interneurons as well as PCs for the development of cortical pathology and behavioral defects^19^. Electrophysiological recordings in various HD mice revealed changes in both excitatory and inhibitory inputs onto layer 2/3 (L2/3) PCs, with a consistent reduction in the frequency of spontaneous inhibitory postsynaptic currents (sIPSCs)^19,25,26^. Despite these insights into the cortical circuit impairments in HD, it has remained unclear how cortical network function is affected *in vivo* during the presymptomatic phase and at disease onset, and which molecular and circuit mechanisms underlie these functional alterations.

Here, we use chronic *in vivo* two-photon calcium imaging in awake, behaving HD mice to monitor the activity of large populations of L2/3 neurons in the M1 area at single-cell resolution. Our imaging experiments reveal an increase in neuronal activity that affects a large fraction of cells and precedes the onset of motor impairments. Proteomic analyses show a pronounced downregulation of synaptic proteins in the cortex, whereas histological findings in HD mouse brains and in human postmortem tissue point to a loss of inhibitory synapses on PCs, suggesting that excitation/inhibition dysbalance plays a role in the cortical dysfunction in HD.

## Results

### Chronic two-photon calcium imaging in the cortex of R6/2 mice

**Figure 1:**
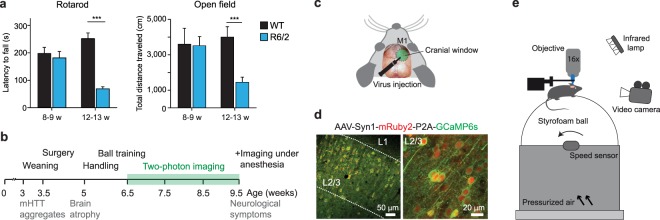
Chronic two-photon calcium imaging in awake R6/2 mice. (**a**) Left: Latency to fall on the accelerating rotarod. Right: Distance traveled in the open field. ***p < 0.001. (**b**) Experimental design and timeline of R6/2 phenotypes. (**c**) Scheme of a cranial window over the M1 cortex. (**d**) Coronal brain section showing AAV1/2-mediated expression of mRuby2 (red) and GCaMP6s (green) in L2/3 neurons in M1. (**e**) Imaging setup. A mouse head-fixed under a two-photon microscope is placed on a spherical treadmill floating on pressurized air. Neuronal activity is monitored through a cranial window. Running behavior is registered by an IR-sensitive video camera and a speed sensor.

We investigated neuronal activity in R6/2 transgenic mice, which express mHTT-exon 1 with a pathological polyQ expansion under the human HTT promoter and are characterized by an early onset and rapid progression of the disease^27–29^. In spite of considerable brain atrophy, no obvious cell loss has been reported in this line until the age of 12 weeks^28,30,31^. Overt neurological symptoms such as tremor, dyskinesia and balance impairment start at 9–11 weeks of age^28^. The exact age of onset of motor deficits varies between different R6/2 colonies and is dependent on the CAG repeat length, which can increase over generations due to genetic instability of the repeats. Several studies have described later onset and overall attenuation of HD-related phenotypes in animals with very high repeat numbers^32,33^. The CAG repeat length in our colony averaged 192 ± 2 repeats and was higher than in the original line (~150 repeats)^28^. We therefore characterized the motor phenotype in our colony by testing the mice on an accelerating rotarod and in the open field. 8 to 9-week-old R6/2 mice showed no significant deficits on the rotarod and normal locomotion in the open field, whereas by the age of 12–13 weeks, they were severely impaired in both tests (WT, 7–10 mice; R6/2, 10 mice; Two-way ANOVA with Bonferroni’s multiple comparison test; Rotarod, 8–9 weeks, p > 0.05; 12–13 weeks, p < 0.001; Open field, 8–9 weeks, p > 0.05; 12–13 weeks, p < 0.001; Fig. 1a). We conclude that the onset of motor defects in our colony occurs after 9 weeks of age and is thus slightly delayed compared to other reports^23,27^, likely due to the expansion of CAG repeats and/or differences in housing conditions.

To study longitudinal changes in neuronal function before the onset of motor impairments, we chronically monitored calcium transients in L2/3 neurons in M1 cortex of R6/2 mice and wildtype (WT) littermates (Fig. 1b). Mice were injected with AAV1/2-Syn1-mRuby2-P2A-GCaMP6s^34^, which allows functional imaging along with morphological labeling of the neurons (Fig. 1c,d). Because of the rapid disease progression in this mouse line, we performed virus injections and cranial window implantations at the age of 3.5 weeks (Fig. 1b). Two-photon imaging sessions of 15 min each were started 3 weeks after the surgery and carried out at weekly intervals between 6.5 and 9.5 weeks of age (Fig. 1b). Populations of 100–200 L2/3 neurons were imaged in awake head-restrained animals during voluntary locomotion in the dark on a spherical treadmill restricted to movement around one axis^35^ (Fig. 1e). Only neurons that could be unambiguously reidentified in all imaging sessions were included in the analysis. The fraction of reidentified cells varied between 65% and 90% per field of view (FOV) and was not significantly different between the genotypes (mean ± SD: WT, 79.3 ± 9.5%; R6/2, 80.6 ± 5.4%; Student’s t-test, p = 0.8254). The experiments ended before the animals developed overt neurological symptoms such as tremor, balance problems or change of posture. During the imaging sessions, mice exhibited spontaneous running behavior, which was recorded by an infrared (IR)-sensitive video camera and/or by an optical mouse sensor (Fig. 1e).

### Increased neuronal activity in HD mice prior to motor impairments

**Figure 2:**
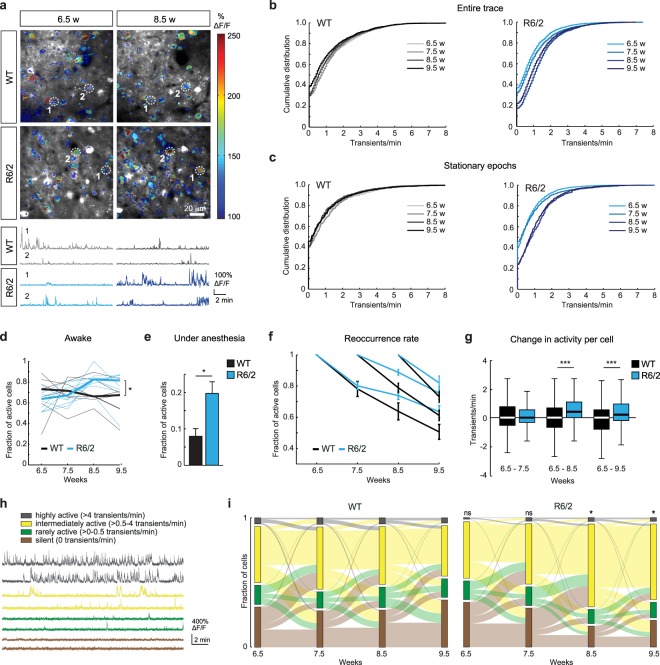
Increased neuronal activity before the onset of motor defects in R6/2 mice. (**a**) Top: Examples of imaged areas in WT and R6/2 mice superimposed by activity maps. Color coding shows normalized maximum activity. Bottom: Calcium traces of neurons marked on the images above. (**b**) Cumulative distributions of calcium transient frequencies at the indicated time points in WT (left) and R6/2 (right) animals. Note a shift in the distribution towards higher frequencies occurring at 8.5 weeks in R6/2 mice. (**c**) Cumulative distributions of calcium transient frequencies during stationary epochs at the indicated time points in WT (left) and R6/2 (right) animals. (**d**) Fraction of active cells at different imaging time points in WT and R6/2 mice. (**e**) Fraction of active cells under 1.5% isoflurane anesthesia at 9.5 weeks. (**f**) Reoccurrence rate of active cells in WT and R6/2 mice. (**g**) Box plots showing activity changes of single cells between the first and later time points. (**h**) Example traces of highly active, intermediately active, rarely active and silent neurons. (**i**) Alluvial plots showing the distribution of imaged cells into four activity categories at each time point (stacked bars), as well as changes between the activity categories over time (stream fields) in WT (left) and R6/2 (right) animals. *p < 0.05, ***p < 0.001.

Calcium transients were observed in many L2/3 neurons, with activity remaining quite stable during the imaging period in WT mice, but increasing on average in R6/2 mutants (Fig. 2a). Accordingly, the distribution of calcium transient frequencies did not change during the imaging period in WT mice, while in R6/2 animals it was shifted towards higher frequencies starting from 8.5 weeks of age (WT, 1612 neurons from 6 mice; R6/2, 2589 neurons from 5 mice; Repeated measures ANOVA, Genotype: F(1, 12597) = 2.01, p = 0.16; Age: F(3, 12597) = 82.91, p < 0.001; Interaction F(3, 12597) = 101.2, p < 0.001; Fig. 2a,b). This effect was also evident when we analyzed the periods during which the animal remained stationary (only experiments with a minimum of 1% stationary time were included in this analysis; WT, 1346 neurons from 6 mice; R6/2, 2589 neurons from 5 mice; Repeated measures ANOVA, Genotype: F(1, 11599) = 0.08, p = 0.78; Age: F(3, 11599) = 73.43, p < 0.001; Interaction F(3, 11599) = 102.66, p < 0.001; Fig. 2c. Of note, the effect also held true for other cutoffs of minimal stationary time), indicating that the elevated activity was independent of locomotion. In agreement with previous studies^28,31^, we did not observe a significant difference in cell numbers in L2/3 of M1 between R6/2 mice and WT littermates at the age of 8 weeks (3 WT and 3 R6/2 mice; Student’s t-test, p = 0.3071; Supplementary Fig. S1), suggesting that the observed changes in network activity are not related to cell death.

Next, we quantified the fraction of active cells (exhibiting at least one calcium transient during an imaging session, see Materials and Methods) at different time points. This analysis revealed a higher fraction of active cells in R6/2 mice from 8.5 weeks onwards (WT, 7 FOVs from 6 mice; R6/2, 10 FOVs from 5 mice; Repeated measures ANOVA, Genotype: F(1, 45) = 1.11, p = 0.31; Age: F(3, 45) = 3.84, p = 0.02; Interaction F(3, 45) = 3.73, p = 0.02; Fig. 2d). After the last awake imaging session, some of the mice were additionally imaged under isoflurane anesthesia. Consistent with our findings in awake animals, we observed more active cells in R6/2 mice than in WT littermates (WT, 6 FOVs from 3 mice; R6/2, 4 FOVs from 2 mice; Wilcoxon rank-sum test, p = 0.038; Fig. 2e). In summary, these data demonstrate elevated neuronal activity in the cortex of HD mice before the onset of neurological and motor impairments.

### Altered dynamics of single-cell activity in HD mice

We next analyzed the reoccurrence rate of active cells, i.e. the fraction of cells that were active at the given imaging time point and remained active in each of the following imaging sessions. In WT mice, the reoccurrence rates of active cells steadily declined throughout the imaging period. In R6/2 animals this decline was also observed, however, it slowed down from 8.5 weeks onwards, resulting in a higher reoccurrence rate compared to WT controls (WT, 7 FOVs from 6 mice; R6/2, 10 FOVs from 5 mice; Repeated measures ANOVA, 6.5 to 9.5 weeks: Genotype: F(1, 45) = 2.61, p = 0.12; Age: F(3, 45) = 130.0, p < 0.001; Interaction F(3, 45) = 3.97, p = 0.01. 7.5 to 9.5 weeks: Genotype: F(1, 30) = 8.59, p = 0.01; Age: F(2, 30) = 94.42, p < 0.001; Interaction F(2, 30) = 5.33, p = 0.01. 8.5 to 9.5 weeks: Genotype: F(1, 15) = 2.01, p = 0.18; Age: F(1, 15) = 94.42, p < 0.001; Interaction F(1, 15) = 2.01, p = 0.18; Fig. 2f).

In addition, we followed activity changes of individual neurons over time. While we observed highly dynamic activity profiles of single cells in both genotypes during the imaging period (Supplementary Fig. S1), there was no net change in the frequency of calcium transients per cell in WT animals at any time point (Fig. 2g). In contrast, we detected an increase in transient frequencies in the majority of cells in R6/2 mice at 8.5 weeks of age (WT, 1612 neurons from 6 mice; R6/2, 2589 neurons from 5 mice; Wilcoxon rank-sum test, WT vs. R6/2, 6.5–7.5 weeks, p > 0.05; 6.5–8.5 weeks, p < 0.001; 6.5–9.5 weeks, p < 0.001; Figs 2g and S1), with the median change per cell between 6.5 and 8.5 weeks amounting to 0.5 transients/min. These data indicate that the increase in activity observed in behaving R6/2 mice is a global effect, due to moderate changes in the activity of many cells, rather than to a small fraction of cells markedly increasing their firing.

We further examined activity levels of single neurons by subdividing them into four activity categories: silent (0 Ca^2+^ transients/min), rarely active (>0–0.5 transients/min), intermediately active (>0.5–4 transients/min), and highly active (>4 transients/min) (Fig. 2h). In the first two imaging sessions, the distribution of cells into these four categories was not significantly different between R6/2 and WT mice (Fig. 2i). However, a clear shift in the distribution was observed in the R6/2 animals between 7.5 and 8.5 weeks of age (WT, 1612 neurons from 6 mice; R6/2, 2589 neurons from 5 mice; Pearson’s Chi-square test, R6/2 vs. WT at 6.5 weeks, p = 0.5347; at 7.5 weeks, p = 0.901; at 8.5 weeks, p = 0.0495; at 9.5 weeks, p = 0.0198), with an increase in the fraction of intermediately active cells, and a reduction in the fraction of silent cells, while the rarely active and the highly active fractions did not change significantly (Fig. 2i). The increase in the intermediately active category in the R6/2 mice at 8.5 weeks was to a large extent due to cells that were classified as silent at 7.5 weeks (26% of silent cells became intermediately active in WT vs. 54% in R6/2; Exact binomial test, p < 0.001). Taken together, these results indicate that the observed increase in activity in R6/2 mice affects a large number of cells in the network and can at least in part be attributed to more silent cells changing into the intermediately active category, as well as more cells staying active between the imaging sessions.

### Increased synchrony in presymptomatic HD mice

**Figure 3:**
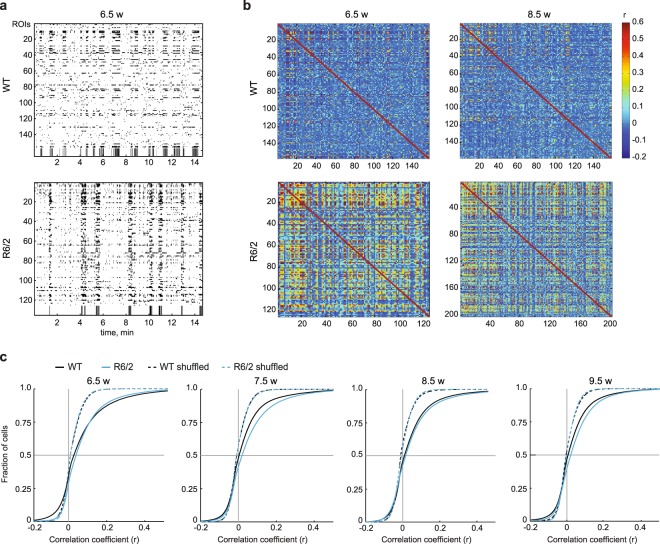
Increased synchrony in R6/2 mice. (**a**) Representative raster plots of single-cell activity in a WT (top) and R6/2 (bottom) mouse during an imaging session at 6.5 weeks of age. Running episodes are depicted in black and stationary periods in white at the bottom of the plots. (**b**) Correlograms from a WT (top) and R6/2 (bottom) mouse shown in (**a**) at 6.5 weeks (left) and 8.5 weeks (right). Only active cells were included in the analysis, resulting in the different numbers of ROIs captured in the correlograms at different time points. (**c**) Cumulative distributions of pairwise correlation values (Pearson’s r) for actual (solid lines) and shuffled (dashed lines) data at different time points. Pairwise correlation analysis was performed on entire traces.

As calcium imaging allows simultaneous observation of activity in hundreds of cells, we next asked whether neuronal synchrony is altered in the cortical network of HD mice. We observed an increase in correlated activity in R6/2 animals compared to WT littermates (Fig. 3a). Analysis of the Pearson’s correlation coefficient (r) for neuronal pairs revealed a small, yet highly significant increase in pairwise correlations in R6/2 mice at all imaging time points (WT, 88580 cell pairs at 6.5 weeks, 114020 cell pairs at 7.5 weeks, 111645 cell pairs at 8.5 weeks, 91655 cell pairs at 9.5 weeks, from 6 mice; R6/2, 143716 cell pairs at 6.5 weeks, 164325 cell pairs at 7.5 weeks, 211011 cell pairs at 8.5 weeks, 222328 cell pairs at 9.5 weeks, from 5 mice; Wilcoxon rank-sum test, WT vs. R6/2, p < 0.001 for all time points; Fig. 3b,c). To exclude the possibility that higher pairwise correlation in HD mice was due solely to the higher frequency of calcium transients in these animals (see Fig. 2b), we also analyzed correlation of shuffled data. For both genotypes, the correlation coefficient of the actual data was clearly higher than that of the shuffled dataset (Wilcoxon rank-sum test, WT vs. WT shuffled, p < 0.001 for all time points; R6/2 vs. R6/2 shuffled, p < 0.001 for all time points; Fig. 3c). These data point to a higher degree of synchrony in the cortical network of presymptomatic R6/2 mice compared to WT animals.

### Downregulation of synaptic proteins in R6/2 cortex prior to motor impairments

**Figure 4:**
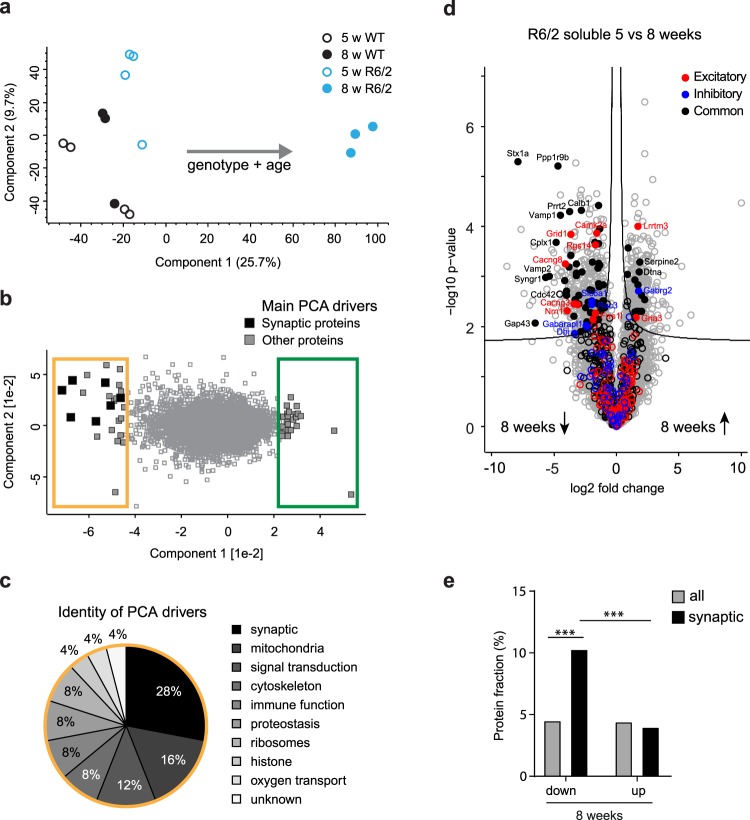
Downregulation of synaptic proteins in R6/2 cortex. (**a**) PCA projections of soluble cortical samples from 5- and 8-week-old R6/2 mice and WT littermates. (**b**) Main PCA drivers of the separation that are downregulated (yellow frame) and upregulated (green frame) in 8-week-old R6/2 mice. Synapse-related proteins are highlighted in black. Main drivers were defined as the top 25 proteins accounting for the separation of samples in the PCA. (**c**) Functional groups of the main PCA drivers downregulated in 8-week-old R6/2 mice (indicated by the yellow frame in **b**). See also Supplementary Table S1. (**d**) Volcano plot showing proteins up- or downregulated in the soluble fraction of 8-week-old compared to 5-week-old R6/2 cortex. Proteins above the curved q-value cutoff line are statistically significantly changed (q < 5%); Excitatory synaptic proteins are highlighted in red, inhibitory in blue, common in black; filled circles indicate significantly changed synaptic proteins. (**e**) Fraction of proteins significantly (q-value < 5%) up- or downregulated in the soluble proteome of the R6/2 compared to WT cortex at 8 weeks. ***p < 0.001. See also Supplementary Table S2.

To gain insight into the potential molecular mechanisms underlying the dysregulated activity in the cortex, we took advantage of the spatiotemporally resolved mass spectrometry (MS)-based proteomic dataset from R6/2 mice and WT littermates that we obtained previously, including changes in the soluble proteome and composition of insoluble mHTT inclusion bodies^36^. For the present study, we focused on cortical samples from 5-week-old (early presymptomatic) and 8-week-old animals (around the age of observed changes in neuronal activity). Principal component analysis (PCA) revealed a clear separation of soluble cortical samples of 8-week-old R6/2 mice from the samples of 5-week-old R6/2 and all WT animals (Fig. 4a). We then asked which proteins accounted for this separation. Interestingly, the largest functional group among the main PCA drivers that were downregulated in 8-week-old R6/2 mice were synapse-related proteins (28%, 7 out of 25 proteins; the fraction of synapse-related proteins in the total proteome was 8%, 651 out of 8455 proteins) (Fig. 4b,c, Supplementary Table S1). In contrast, no synaptic proteins were found among the main PCA drivers upregulated in R6/2 mice (0 out of 25; Fig. 4b).

While there were no major differences between the soluble cortical proteome of R6/2 and WT mice at 5 weeks, we observed marked changes in the R6/2 cortex at 8 weeks of age (Fig. 4d and Supplementary Table S2), consistent with the data from other brain regions^36^. Moreover, there was a pronounced decrease in synaptic protein levels (Fig. 4d,e). Importantly, the fraction of downregulated synaptic proteins (10.3%, 67 out of 651 proteins) was significantly higher than the upregulated fraction (4.0%, 26 out of 651 proteins) (Fisher’s exact test, p < 0.001; Fig. 4e and Supplementary Table S2), while the fractions of proteins that were down- or upregulated in the whole proteome were not significantly different (4.5% downregulated, 382 out of 8456 proteins; 4.4% upregulated, 375 out of 8456 proteins, Fisher’s exact test, p = 0.824; Fig. 4e and Supplementary Table S2). This finding points towards a specific loss of synaptic proteins in HD mice that is not merely due to the general remodeling of the whole proteome. Among the downregulated proteins, we found both excitatory and inhibitory synaptic markers, along with proteins common to both types of synapses (Fig. 4d). We next asked if the downregulation of synaptic proteins might be due to their sequestration within mHTT inclusions. Out of 66 synaptic proteins reduced in the soluble fraction at 8 weeks, 30 were detected in the insoluble proteome, which largely consists of mHTT inclusions. Remarkably, none of the detected proteins that were reduced in the soluble fraction (synaptic or non-synaptic) were found to be significantly altered in the insoluble fraction of R6/2 mice (Supplementary Fig. S2 and Supplementary Table S3). Sequestration within mHTT inclusions is therefore likely not a major mechanism of protein decrease at an early stage of HD progression. This is distinct from the situation in 12-week-old R6/2 brains, where many proteins downregulated in the soluble pool are enriched in the insoluble material, indicative of their recruitment to mHTT inclusion bodies^36^. In summary, our proteomic results reveal a broad downregulation of synaptic components already before appearance of motor dysfunction.

### Loss of inhibitory synapses on PCs

Lower levels of synaptic proteins in the R6/2 mice might reflect a loss of synapses. A decrease in excitatory synapses in the R6/2 cortex at an advanced disease stage has been reported previously^24^. However, it has remained unclear whether inhibitory contacts are also affected and whether synapse numbers are already reduced before motor symptoms develop. We therefore performed immunostainings for excitatory and inhibitory pre- and postsynaptic markers in L2/3 of the M1 cortex of 8-week-old animals and quantified densities of opposing VGlut1/2; PSD-95 puncta as putative excitatory synapses and opposing VGAT; Gephyrin puncta as putative inhibitory synapses (Supplementary Fig. S2). These experiments did not reveal significant changes in total excitatory or inhibitory synapse densities at 8 weeks (3 FOVs per mouse from 5 WT and 4 R6/2 mice; Student’s t-test; excitatory synapses, p = 0.8279; inhibitory synapses, p = 0.6773; Supplementary Fig. S2).

**Figure 5:**
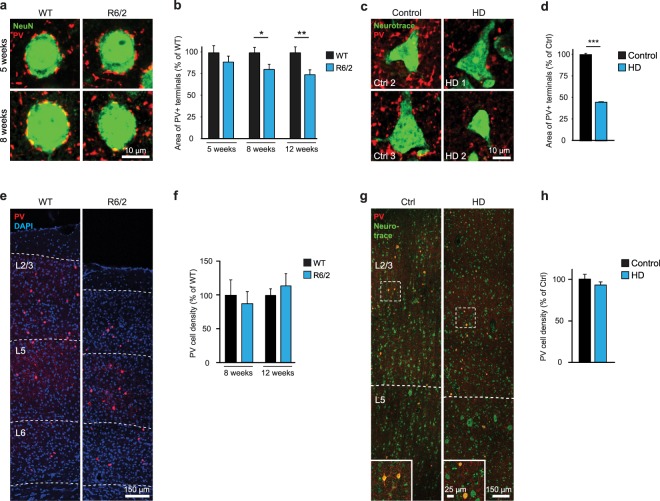
Reduction in inhibitory synaptic terminals on PCs in R6/2 mice and HD patients. (**a,b**) Representative images (**a**) and quantification (**b**) of PV+ synaptic terminals (red) on NeuN-labeled PC cell bodies (green) in L2/3 of M1 cortex from R6/2 mice and WT controls. (**c,d**) Representative images (**c**) and quantification (**d**) of PV+ synaptic terminals (red) on Neurotrace-labeled PC cell bodies (green) in L2/3 of M1 cortex from HD autopsy cases and controls. (**e**) Representative images of PV+ cells in M1 cortex of 8-week-old WT and R6/2 mice. Nuclei were counterstained with DAPI. Dashed lines denote borders between cortical layers. (**f**) Quantification of PV+ cell densities in M1 cortex of WT and R6/2 mice. (**g**) Representative images of PV+ cells in M1 cortex of HD cases and controls. Neuronal cell bodies are labeled with Neurotrace. Dashed lines denote borders between cortical layers. Insets show a higher magnification of the areas marked by dashed boxes. (**h**) Quantification of PV+ cell densities in M1 cortex of HD cases and controls. *p < 0.05; **p < 0.01; ***p < 0.001.

We next focused on a specific subpopulation of inhibitory synapses, parvalbumin (PV)-positive terminals on PCs, as PV+ GABAergic inputs are known to provide the strongest source of inhibition onto PCs^37,38^. The area of PV+ puncta surrounding NeuN-labeled PC cell bodies in L2/3 of the M1 cortex of R6/2 mice was unchanged at 5 weeks of age, but became significantly reduced at 8 weeks, and further decreased at 12 weeks (WT, 74 PCs from 5 mice, 63 PCs from 5 mice, and 45 PCs from 3 mice for 5, 8 and 12 weeks of age, respectively; R6/2, 74 PCs from 5 mice, 58 PCs from 4 mice and 45 PCs from 3 mice for 5, 8 and 12 weeks of age, respectively; Student’s t-test, 5 weeks, p = 0.3131; 8 weeks, p = 0.0257; 12 weeks, p = 0.0047; Fig. 5a,b). Double immunostainig for PV and the inhibitory presynaptic protein VGAT revealed a clear colocalization of the two markers around PC cell bodies (Fig. S2), indicating that perisomatic PV+ puncta truly represent inhibitory synaptic terminals. To validate these histological findings in human disease cases, we performed immunostainings for PV+ terminals in postmortem brain tissue of HD patients and age-matched controls (Supplementary Table S4). Consistent with the results from R6/2 mice, we observed a marked reduction in the area of PV+ puncta around L2/3 PCs in the primary motor cortex (Ctrl, 69 PCs from 3 brains; HD, 75 PCs from 3 brains; Student’s t-test, p < 0.001; Fig. 5c,d). To find out whether the reduction in PV+ inputs is due to cell loss in this interneuron population, we also analyzed the density of PV+ cell bodies. As L2/3 PCs were shown to receive both local as well as translaminar PV+ inputs^39^, PV+ cell density was quantified throughout the cortex. We did not observe significant changes in PV+ cell density in 8-week-old or 12-week-old R6/2 mice (8 weeks, 5 WT and 4 R6/2 mice; 12 weeks, 3 WT and 3 R6/2 mice; Student’s t-test, 8 weeks, p = 0.3997; 12 weeks, p = 0.2986; Fig. 5e,f), or in HD patients (3 controls and 3 HD cases; Student’s t-test, p = 0.3490; Fig. 5g,h), suggesting that the decrease in PV+ teminals is not a result of lower PV+ cell numbers. In summary, loss of GABAergic inputs onto PCs occurs both in R6/2 mice and in human HD cases, suggesting that weakened inhibition might at least partially explain dysregulated cortical activity in HD.

## Discussion

Using chronic *in vivo* imaging in awake animals, we demonstrate defects of cortical network function in HD mice, with an overall increase in activity, higher fraction of active cells, and increased pairwise correlations. Our results extend previous observations of dysregulated neuronal activity and cortical hyperexcitability in HD rodent models detected by electrophysiology in slices and *in vivo*^8,25,40^. Moreover, a recent two-photon imaging study, conducted in the visual cortex of anesthetized Hdh150 knock-in mice, also described an increase in activity and synchronicity in premanifest HD animals^41^. While our findings are in agreement with these studies, our chronic imaging approach enabled us for the first time to observe cortical network changes in awake animals at a single-cell resolution, and to pinpoint the exact timing of these changes. Remarkably, most of the alterations we observed occurred rather abruptly at the age of 8.5 weeks, before the appearance of motor defects. These impairments of cortical activity might therefore critically contribute to the onset of motor dysfunction. In addition, increased synchrony was already detected at 6.5 weeks, the earliest age examined, suggesting that subtle changes in cortical network function already happen at an early presymptomatic stage.

The R6/2 transgenic fragment model used in this study was chosen because of its well-characterized phenotype and rapid disease progression. Furthermore, it shows extensive similarities with more recent models expressing full-length mHTT in terms of cortical pathology and electrophysiology^25,42^. Nevertheless, it will be important to validate our findings in a full-length HD model in the future.

Dysregulation of cortical activity observed in HD mice might in turn lead to profound changes of neuronal function in the striatum. Early in HD progression, hyperactivity of striatal neurons was described in R6/2 mice^43^, and elevated glutamate levels were detected in the striatum of HD patients^44^. These alterations could in part be due to increased activation of the striatum by cortical inputs. Remarkably, removal of cortical afferents resulted in amelioration of HD phenotypes in R6/2 mice^45^, and expression of mHTT in cortical neurons was necessary and sufficient to cause functional impairments of the striatal compartment in a corticostriatal network reconstructed *in vitro*^46^. It remains to be tested whether restoration of normal cortical activity levels, e.g. with the help of chemogenetic tools, would also be sufficient to rescue or delay HD symptoms. It should be noted that the majority of corticostriatal afferents originate from L5 PCs, however, L2/3 cells imaged here provide the main source of input to L5 PCs^47^ and are therefore likely to critically influence corticostriatal communication. In future experiments it will be important to also assess the function of L5 neurons directly.

The excessive activity observed in R6/2 mice was broadly distributed across the network, with a majority of cells showing a moderate increase in calcium transient frequency, suggesting that HD-related neurodegenerative processes have a global impact on the cortical network. Interestingly, another *in vivo* imaging study conducted in anesthetized Hdh150 mice revealed an increase in the fraction of hyperactive cells in the visual cortex at an early, “very far from disease onset” stage^41^. This is in contrast to our finding of a mild and broadly distributed increase in activity without the appearance of hyperactive neurons. This discrepancy might be due to the different disease stages, cortical areas and mouse models used, as well as methodological differences between the studies, such as the usage of different calcium indicators and imaging in awake vs. anesthetized animals. To reconcile these observations, it would be necessary to perform chronic imaging in HD mice during a broader time period from very early presymptomatic to symptomatic disease stages. We furthermore detected altered activity dynamics at single-cell level, with more neurons remaining active between imaging sessions, as well as more cells changing from silent to intermediately active. Highly dynamic activity patterns that are characteristic of the motor cortex are believed to be important for motor learning^48^. Of note, motor learning impairments have been described in HD mouse models^49,50^ as well as HD patients^51^. In future studies, it will be interesting to investigate the possible role of cortical activity in these behavioral changes by combining *in vivo* microscopy with a learning task.

What might be the molecular link between expression of mHTT and defects of neuronal communication in the cortex? Our proteomic analyses demonstrated a major downregulation of synaptic proteins in R6/2 mice. In addition to confirming reductions in certain synaptic proteins shown previously in HD mice and human brain samples at advanced disease stages^52–55^, our dataset reveals a broad downregulation of multiple synaptic components that occurs already before any motor abnormalities can be detected. Moreover, while the loss of general and excitatory synaptic markers in HD cortex is well documented^24,52,55^, we find that inhibitory synaptic proteins are singificantly affected as well. This is in agreement with recent transcriptomic data showing downregulation of genes involved in GABA metabolism and signaling in iPSC-derived neural cultures from HD patients^56^. Surprisingly, the majority of synaptic proteins that were downregulated in the soluble fraction were not altered in the insoluble material at 8 weeks of age, arguing against sequestration as a major mechanism of synaptic protein depletion at this disease stage^23,36^. Synaptic defects in HD might result to some extent from loss of native HTT function, as HTT is required for normal synapse development in the cortex^57^. Another likely mechanism of synaptic protein reduction might be transcriptional dysregulation, as genes related to synaptic signaling were among the most prominent dysregulated gene clusters in transcriptomic analyses of HD mice and human iPSC-derived neural cultures^56,58,59^.

In addition to the reduction in synaptic proteins detected by mass spectrometry, our histological analyses revealed a reduction in PV+ inhibitory synaptic contacts on PCs in R6/2 mice in the absence of PV+ cell loss, which was confirmed in human HD autopsy cases. It should be kept in mind that postmortem HD brain tissue represents an advanced disease stage. Nevertheless, the similarity of histological findings in R6/2 mice and HD patients illustrates the relevance of our results for the human disease. While the exact causal link between synaptic defects and the observed cortical network disturbances remains to be investigated, our findings suggest that abnormalities of cortical function in HD could at least in part be explained by a disturbed excitation/inhibition balance. This hypothesis is also in agreement with the increased synchrony in the cortex of HD mice observed by us and others^41^, as correlated firing in cortical circuits is controlled by inhibitory interneurons^60^. Reduced frequency of inhibitory postsynaptic currents has been described in L2/3 PCs in the motor and somatosensory cortex in several HD mouse models at a symptomatic stage^19,25,26^. Remarkably, human studies with the use of transcranial magnetic stimulation revealed abnormal cortical excitability due to dysfunctional inhibition in presymptomatic and early-stage HD patients^16,61^, emphasizing the translational value of our findings. Impairments in the function of inhibitory neurons have also been suggested to underlie neural circuit defects in mouse models of Alzheimer’s disease^62,63^ and may therefore represent a common phenomenon in various neurodegenerative disorders. Taken together, our chronic single-cell resolution imaging approach, in combination with quantitative proteomic analyses and histological experiments, points towards disturbed cortical excitation/inhibition balance at the presymptomatic stage as a key mechanism in HD pathogenesis.

## Methods

### Transgenic mice

R6/2 mice^28^ transgenic for the 5′ end of the human *huntingtin* gene were maintained by crossing R6/2 males to F1 C57Bl6/CBA females. Animals were kept in a specific pathogen-free animal facility with free access to food and water. The presence of the transgene was verified by PCR with the following primers: forward, 5′-CCGCTCAGGTTCTGCTTTTA-3′, reverse, 5′-TGGAAGGACTTGAGGGACTC-3′. CAG repeat length was determined by Laragen. Female R6/2 mice and WT littermates were used in all experiments. After surgery, mice were kept in inverted 14–10 h light-dark cycle. All animal experiments were approved by the Government of Upper Bavaria (animal protocols 55.2-1-54-2532-168-2014, 55.2-1-54-2532-19-2015), and all the methods were performed in accordance with the relevant guidelines and regulations.

### Behavioral tests

#### Rotarod

Rotarod test was performed on a Rota-Rod NG (Ugo Basile). Mice were first trained on two consecutive days for 300 s at 5 rpm, and then tested with acceleration from 5 to 40 rpm over a 300 s period. Latency to fall was recorded on three trials separated by 15 min resting periods, and the average value was taken for analysis.

#### Open field

Locomotor activity in the open field was assessed one hour after the beginning of the dark cycle. Mice were placed into a custom-made 40 × 40 cm arena, and total distance traveled was recorded for 10 min. The floor of the chamber was washed between the trials to minimize any olfactory cues that could affect exploratory behavior.

### Virus injection and cranial window surgery

For *in vivo* calcium imaging, an adeno-associated virus (AAV1/2) containing the genetically encoded calcium indicator GCaMP6s^64^ and the structural marker mRuby2^65^ under the control of the human synapsin-1 promoter was used to label cortical neurons^34^. Intracerebral injections of AAV and cranial window implantation were performed within the same surgery in 3.5-week-old mice deeply anesthetized with an intraperitoneal (i.p.) injection of ketamine/xylazine (130 and 10 mg kg^−1^ body weight, respectively). The analgesic carprofen (5 mg  kg^−1^ body weight, subcutaneously) and the anti-inflammatory drug dexamethasone (10 mg kg^−1^, i.p.) were administered shortly before surgery. To increase viral uptake and spread, mannitol (20% solution; 30 ml kg^−1^ body weight, i.p.) was applied 20 min prior to virus injection^66^. During surgery, the virus (titer: ~10^12^ infecting units per ml) was injected into L2/3 of M1 cortex (3 injection sites with stepwise 300 nl injections at 150, 200 and 250 µm depth). A cranial window was implanted over the right cortical hemisphere as previously described^67^. Briefly, a circular piece of skull (4 mm in diameter) was removed over the fore- and hindlimb area of M1 (position: 1.3 mm lateral and 1.0 mm anterior to bregma) using a dental drill (Foredom). A round coverslip (VWR; d = 4 mm) was glued to the skull using histoacryl glue (B.Braun) and dental acrylic cement (Kerr Vertise Flow). After surgery, mice received a subcutaneous injection of the antibiotic cefotaxime (60 mg kg^−1^) and were placed in a warm environment for recovery. After 10 days, a small custom-made metal bar (1 cm × 3 cm; 0.06 g) with a round opening was glued onto the coverslip with dental acrylic cement to allow for stable head fixation under the objective and repeated repositioning of mice during subsequent imaging sessions. Imaging began after a 21-day resting period after surgery.

### Handling and ball training

At the age of 5 weeks, mice were handled on 5 consecutive days for 10 min until they were familiarized with the trainer and routinely ran from hand to hand. In the subsequent ball training mice got adjusted to the experimental setup and head fixation. Ball training sessions that were repeated on 3 consecutive days were set in the dark with an IR light source and lasted for 30 min. Mice were head-fixed by the metal bar to a custom-made holder and placed onto a styrofoam ball (d = 20 cm) that floated on pressurized air^35^. After the third session, mice had adjusted to the head fixation and showed alternating running and resting behavior.

### Two-photon calcium imaging in behaving mice

During the experiment, the same conditions as during ball training were applied. Mouse behavior was tracked at 15 Hz with an IR-sensitive video camera (USB 2.0, 1/3″CMOS, 744 × 480 pixel; 8 mm M0814MP2 1.4–16 C, 2/3″, megapixel c-mount objective; TIS) and custom software (Input Controller, TIS). In addition, to track ball motion, a computer gaming mouse (G500S, Logitech) was positioned along the ball axis, controlled by a raspberry pi3 and custom written scripts using PuTTY to count ball rotation events (1000 counts/s). To synchronize *in vivo* two-photon imaging, behavioral video recording and ball speed measurements, a 900 s lasting TTL pulse (5 V) was sent to relevant hardware using Matlab (Mathworks). *In vivo* calcium imaging was performed with an upright multiphoton microscope (Bergamo II, Thorlabs) equipped with a Ti:Sapphire laser with dual beam (InSight DeepSee, Spectra Physics), a 8 kHz galvo/resonant scanner and a 16x, 0.8 NA water immersion objective (Nikon). The laser intensities were modulated with Pockels cells (Conoptics). The following wavelengths and emission filters were used to simultaneously image the two fluorophores: 920 nm/525 ± 25 nm (GCaMP6s) and 1040 nm/607 ± 35 nm (mRuby2). ThorImage 2.4 software (Thorlabs) was used for microscope control and image acquisition. To measure neuronal activity, time series images of selected positions/FOVs with bright expression of the calcium indicator were acquired at 10 Hz for a total duration of 900 s. For each mouse, two FOVs were recorded per imaging time point at the depth of 150–350 μm. For each FOV, an image of the blood vessel map was acquired under epifluorescence to ensure return to the same position in serial experiments. In addition, the information about XYZ-coordinates provided by the microscope stage was documented for each image position. The structural marker mRuby2 was used to precisely adjust the FOV in the z-plane. After imaging, the animal was returned back to its housing cage for rest. At the last imaging time point, in some of the mice the same FOVs were additionally imaged for 300 s during isoflurane (1.5%) anesthesia.

### Time series image processing and data analysis

Image analysis was performed with ImageJ (NIH) and Matlab software using custom written procedures. First, full frame images were registered and motion corrected in ImageJ using the moco plugin^68^. Next, regions of interest (ROIs) were drawn manually around individual somata based on both maximum and mean intensity projections of all frames. For neighboring cells with direct contact, pixels containing signals from both cells were excluded from the selection. For each imaging time point, the ROIs were visually inspected in the GCaMP and mRuby channel to ensure that the same cells were analyzed throughout the imaging period. The fluorescence intensity of all pixels inside each ROI was averaged and mean values were imported into Matlab for further processing as described previously^69^. To account for neuropil contamination, the following correction method was applied: the initial ROI was fitted with an ellipse and this ellipse was stretched by 6 pixels. All pixels of the initial ROI, as well as those of neighboring ROIs were excluded from the resulting larger ellipse. Next, the corrected ROI signal was computed as follows: F_ROI_comp_ = F_ROI_ − 0.7 × (F_neuropil_ − median (F_neuropil_)), with F_ROI_comp_ representing the neuropil compensated fluorescence of the ROI, F_ROI_ referring to the fluorescence signal of the initial ROI selection and F_neuropil_ to the signal stemming from the neuropil^69^. To estimate the baseline level (F_0_), each fluorescence trace was divided by the median of all values smaller than the 70^th^ percentile of the entire trace, which reflects the baseline well as judged by visual inspection. Cells were classified as active in a particular experiment if they crossed a threshold of baseline +3 × standard deviation (SD) of the ∆F/F trace at least once for a minimum of 10 consecutive frames (1 s). The degree of synchrony between neurons was computed based on entire binarized calcium traces. To this end, the ∆F/F traces of active cells were low pass filtered at 0.3 Hz, smoothed across 4 frames and subsequently thresholded at 2 × SD of the noise band of the original trace. The Pearson’s correlation coefficient (r) for each ROI pair within a FOV was then computed. Shuffled data was generated by circularly shifting the calcium trace of each ROI by a random value.

Video sequences acquired for behavioral tracking were analyzed in EthoVision (Noldus), using the activity analysis tool. Briefly, a ROI was drawn manually around the forepaws. Changes in pixels induced by forepaw movement were registered as activity change and plotted over time by determined algorithms. For direct comparison of video and imaging data at equal frame numbers, the 15 Hz videos were reduced to 10 Hz using the signal.resample function from Scipy and imported into Matlab for further analysis.

### MS data analysis

All data was obtained from the proteomic dataset published by Hosp *et al*.^36^. Log2-transformed label free quantification (LFQ) protein intensity values were used for quantitative analysis. Bioinformatic analyses were conducted with the Perseus software package^70^ using version 1.6.1.3 for Volcano plots and 1.5.2.11 for all other analyses. The cutoff for statistical significance was a q-value of 5% using the permutation-based decoy model for false discovery rate (FDR) control in multiple testing within the Perseus package. The s0-parameter weighing the effect size of protein changes in Volcano plot q-value calculation was kept at the default value of 0.1. Annotations were based on GOCC, GOBP, GOMF, CORUM, uniprot protein family, uniprot interaction partner, Pfam domains and KEGG pathway annotations with the exception of the main PCA drivers, which were complemented by manual annotations based on literature searches.

### Immunofluorescent staining and confocal microscopy

Mice were transcardially perfused with phosphate-buffered saline (PBS) followed by 4% paraformaldehyde (PFA) in PBS. Brains were dissected out, post-fixed in 4% PFA at 4 °C for 48 h, and coronal brain sections (70 μm) were cut on a microtome (VT 1000 S, Leica). Free-floating sections were permeabilized in 0.5% TritonX-100 in PBS for 30 min and blocked in 5% normal donkey serum, 0.2% bovine serum albumin (BSA), 0.2% glycine, 0.2% lysine, 0.02% sodium azide in PBS for 2 h at room temperature, followed by overnight exposure to primary antibodies in 0.3% TritonX-100, 2% BSA, 0.02% sodium azide in PBS. The following primary antibodies were used: rabbit anti-PV (Abcam), mouse anti-NeuN (Millipore), guinea-pig anti-VGlut1 (Millipore), guinea-pig anti-vGlut2 (Millipore), mouse anti-PSD-95 (Sigma), mouse anti-Gephyrin (Synaptic Systems), rabbit anti-VGAT (Synaptic Systems), and mouse anti-VGAT (Synaptic Systems). After several washes with PBS, sections were incubated in corresponding Alexa secondary antibodies (Invitrogen) diluted 1:300 for 2 h followed by 10 min DAPI (Sigma) staining, several PBS washes and mounting with fluorescent mounting medium (DAKO). Fluorescence images were acquired with a Leica TCS SP8 confocal microscope using a 63x, 1.40 NA oil immersion objective (Leica). Image analysis was performed blindly. Cell counts were conducted on DAPI images using a custom-written macro in Cell Profiler. For analysis of synapse densities, images of pre- and postsynaptic stainings were converted into binary masks in ImageJ. Puncta of 2 pixels or less were excluded from further analysis. The coordinates of the remaining puncta were extracted using the “analyze particles” function. The distance of every presynaptic particle to every postsynaptic particle was then calculated using the Pythagorean equation and matrix calculations in R. A distance of 1 μm or less was counted as a synapse. For PV puncta quantification, the PC circumference was traced manually and measured with ImageJ. The area of the cell body and the surrounding PV staining were extracted and the PV-positive pixels counted using a custom written macro. The area of PV terminals around the cell body was normalized to the cell body perimeter.

### Patient material

5 µm paraffin sections from the primary motor cortex of 3 HD autopsy cases and 3 age-matched controls were obtained from Neurobiobank Munich, Ludwig-Maximilians University Munich. Informed consent was available for all cases. The experiments were approved by the ethics committee of Ludwig-Maximilians University Munich, and performed in accordance with the relevant guidelines and regulations. All HD cases were symptomatic. Demographic information is provided in Supplementary Table S4. Sections were deparaffinized in Xylene twice for 5 min, hydrated in a decreasing ethanol concentration series, and transferred to warm tap water for 5 min. Antigen retrieval was conducted in boiling Tris-EDTA buffer (10 mM Tris, 1 mM EDTA, 0.05% Tween 20, pH 9.0) for 15 min. Slides were then transferred to tap water and blocked with BLOXALL Blocking Solution (Vector Laboratories) for 10 min and subsequently with 2.5% horse serum for 30 min. The following primary antibodies were used: rabbit anti-PV, 1:250 (Abcam) and mouse anti-CamK2α, 1:250 (Abcam). After several washes with PBS, sections were incubated in corresponding Alexa secondary antibodies (Invitrogen) diluted 1:300, and Neurotrace, 1:500 (Life technologies) for 2 h followed by 10 min DAPI (Sigma) staining, several PBS washes and mounting with fluorescent mounting medium (DAKO). Fluorescence images were acquired with a Leica TCS SP8 confocal microscope using a 63x, 1.40 NA oil immersion objective (Leica). PCs in L2/3 were identified by their triangular shape, the presence of a nucleolus seen with Neurotrace, and/or through Camk2α-positive staining. Image analysis was performed with ImageJ and R. For PV+ puncta quantification, the circumference of the pyramidal neuron was traced manually, dilated by 1.5 µm and measured with ImageJ. The area of the cell body and the surrounding PV staining was extracted and the PV-positive pixels counted using a custom-written macro. The area of PV+ terminals around the cell body was normalized to the cell body perimeter.

### Statistical analysis

Data were analyzed in a blinded manner. Data are expressed as mean ± SEM unless indicated otherwise, with p < 0.05 defining differences as statistically significant (*p < 0.05; **p < 0.01; ***p < 0.001; n.s. - not significant).

## Supplementary information

Supplementary information

Supplementary table S1

Supplementary table S2

Supplementary table S3

## Data Availability

The data generated during this study are available from the corresponding authors upon reasonable request.
