# Supplementary material for: Cortical circuit alterations precede motor impairments in Huntington’s disease mice
Source: Sci Rep. 2019 Apr 29;9:6634. doi: 10.1038/s41598-019-43024-w (PMC6488584; doi:10.1038/s41598-019-43024-w)
Supplement: Supplementary file 1 — Supplementary information [file 41598_2019_43024_MOESM1_ESM.pdf]

Supplementary Information

Cortical circuit alterations precede motor impairments in Huntington's disease mice

Johanna Burgold, Elena Katharina Schulz-Trieglaff, Kerstin Voelkl, Sara Gutiérrez-Ángel, Jakob Maximilian Bader, Fabian Hosp, Matthias Mann, Thomas Arzberger, Rüdiger Klein, Sabine Liebscher and Irina Dudanova

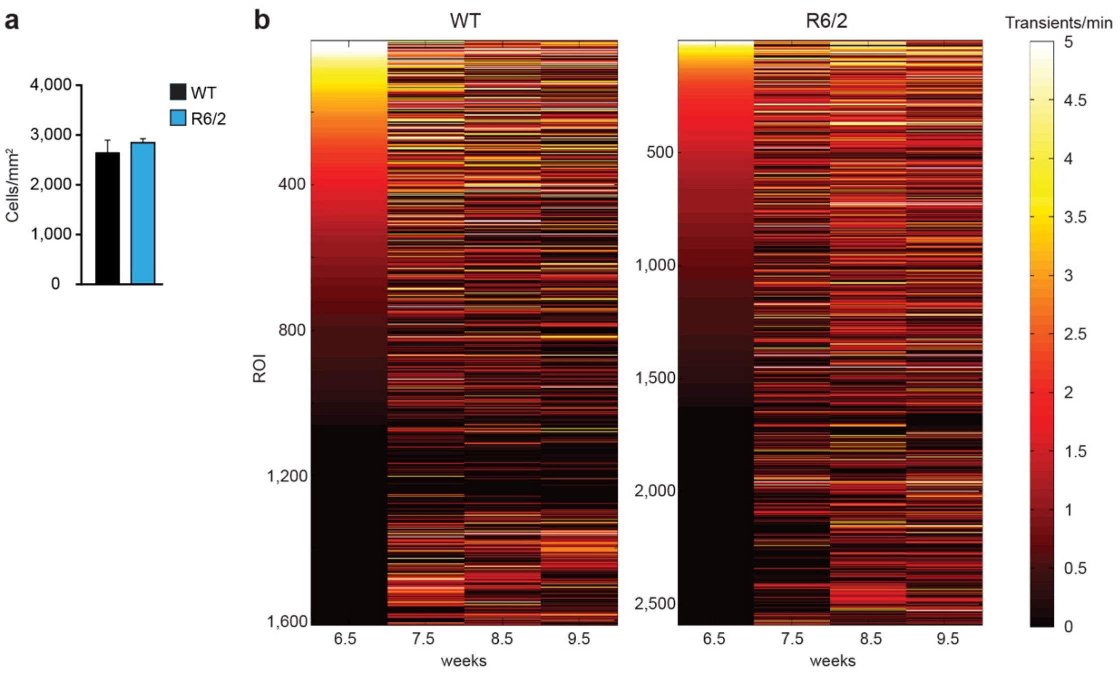

Supplementary Figure S1. Analysis of cell numbers and heat maps of single-cell activity. (**a**) Quantification of cell numbers in L2/3 of M1 cortex in 8-week-old R6/2 and WT mice. (**b**) Heat maps of activity of each individual imaged neuron tracked over time in WT (left) and R6/2 (right) mice. Cells were sorted by activity at 6.5 weeks.

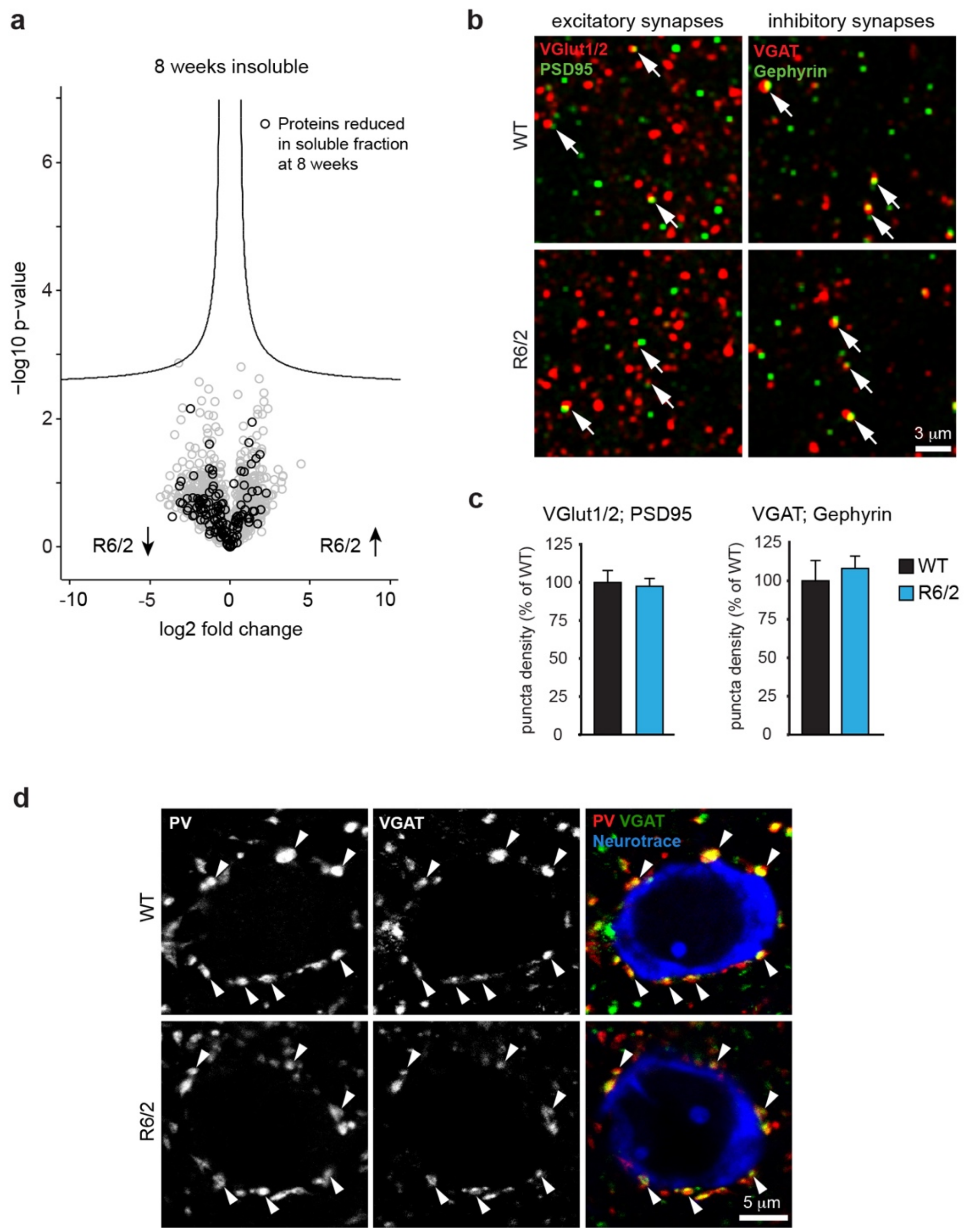

Supplementary Figure S2. Analysis of the insoluble cortical proteome and synapse densities. (**a**) Volcano plot of the cortical insoluble fraction at 8 weeks. Proteins significantly downregulated in the soluble proteome of R6/2 mice at this age (i.e. proteins above the curved q-value cutoff line on the left side of the graph in Fig. 4d) are highlighted in black. Note that none of these proteins are significantly changed in the insoluble proteome. Curved cutoff lines show statistical significance of q < 5%. See also Supplementary Table S3. (**b**) Representative images of excitatory (left) and inhibitory (right) synapses in L2/3 of the M1 area in 8-week-old WT (top) and R6/2 (bottom) mice. Synapses were identified by the overlap or close apposition (arrows) of a presynaptic (red) and postsynaptic (green) marker. (**c**) Quantification of excitatory (left) and inhibitory (right) synapse densities in WT and R6/2 mice. (**d**)

Representative examples of PV and VGAT co-staining in the perisomatic region of L2/3 PCs in M1 of 8-week-old WT (top) and R6/2 (bottom) mice. Note extensive colocalization (arrowheads) of PV puncta with VGAT labeling around the PC soma. Similar results were obtained from 3 WT and 3 R6/2 mice.

Supplementary Table S4. Demographic data of HD patients and controls.

Case no.	Sex	Age at death
HD 1	Male	70
HD 2	Male	60
HD 3	Male	56
Ctrl 1	Male	62
Ctrl 2	Male	70
Ctrl 3	Female	60
